# Correction: Role of Positron Emission Tomography/Computed Tomography (PET/CT) in Detecting Metachronous Primary Tumors: Diagnostic and Clinical Implications From a Single-Patient Case

**DOI:** 10.7759/cureus.c465

**Published:** 2026-07-24

**Authors:** Anmar Z Alharganee, Zahraa A Mohammed, Alhussein Ashraf Kadhim, Marwah Abdulrahman

**Affiliations:** 1 Department of Medical Oncology, Oncology Teaching Hospital, Baghdad Medical City, Baghdad, IRQ; 2 Department of Medical Oncology, Baghdad Medical City, Baghdad, IRQ; 3 Department of Nuclear Medicine and PET/CT, King Hussein Cancer Center (KHCC), Amman, JOR

This article has been corrected to update the case details in Table 1 and remove an unused reference.

